# Risk factors of postoperative hydrocephalus following decompressive craniectomy for spontaneous intracranial hemorrhages and intraventricular hemorrhage

**DOI:** 10.1097/MD.0000000000031086

**Published:** 2022-10-14

**Authors:** Yi-Chieh Wu, Hsiang-Chih Liao, Jang-Chun Lin, Yu-Ching Chou, Da-Tong Ju, Dueng-Yuan Hueng, Chi-Tun Tang, Kuan-Yin Tseng, Kuan-Nien Chou, Bon-Jour Lin, Shao-Wei Feng, Yi- An Chen, Ming-Hsuan Chung, Peng-Wei Wang, Wei-Hsiu Liu

**Affiliations:** a Department of Neurological Surgery, Tri-Service General Hospital and National Defense Medical Center, Taipei, Taiwan; b Department of Radiation Oncology, Shuang Ho Hospital, Taipei Medical University, Taipei, Taiwan; c Department of Radiology, School of Medicine, College of Medicine, Taipei Medical University, Taipei, Taiwan; d School of Public Health, National Defense Medical Center, Taipei, Taiwan; e Department of Surgery, School of Medicine, National Defense Medical Center, Taipei, Taiwan.

**Keywords:** decompressive craniectomy, postoperative hydrocephalus, spontaneous intracranial hemorrhage

## Abstract

Hydrocephalus is a complication of spontaneous intracerebral hemorrhage; however, its predictive relationship with hydrocephalus in this patient cohort is not understood. Here, we evaluated the incidence and risk factors of hydrocephalus after craniectomy.

**Methods:**

Retrospectively studied data from 39 patients in the same hospital from 2016/01 to 2020/12 and analyzed risk factors for hydrocephalus. The clinical data recorded included patient age, sex, timing of surgery, initial Glasgow Coma Scale score, intracerebral hemorrhage (ICH) score, alcohol consumption, cigarette smoking, medical comorbidity, and blood data. Predictors of patient outcomes were determined using Student t test, chi-square test, and logistic regression.

**Results:**

We recruited 39 patients with cerebral herniation who underwent craniectomy for spontaneous supratentorial hemorrhage. Persistent hydrocephalus was observed in 17 patients. The development of hydrocephalus was significantly associated with the timing of operation, cigarette smoking, and alcohol consumption according to the Student t test and chi-square test. Univariate and multivariate analyses suggested that postoperative hydrocephalus was significantly associated with the timing of surgery (*P* = .031) and cigarette smoking (*P* = .041).

**Discussion:**

The incidence of hydrocephalus in patients who underwent delayed operation (more than 4 hours) was lower than that in patients who underwent an operation after less than 4 hours. nonsmoking groups also have lower incidence of hydrocephalus. Among patients who suffered from spontaneous supratentorial hemorrhage and need to receive emergent craniectomy, physicians should be reminded that postoperative hydrocephalus followed by ventriculoperitoneal shunting may be necessary in the future.

## 1. Introduction

Spontaneous non-traumatic intracranial hemorrhage (ICH) is the second most common form of stroke (approximately 15–30% of all strokes). It is also the deadliest disease and has high morbidity and mortality rates. The main causes of spontaneous ICH include poorly controlled hypertension, acutely increased cerebral blood flow, vascular anomalies, and coagulopathies, such as antiplatelet agents. Neurological deterioration after the initial hemorrhage is usually due to a combination of rebleeding, cerebral edema, seizures, increased intracranial pressure (ICP), and hydrocephalus ^[1,2]^; these are related to poor functional outcomes and morbidity.

The initial management of spontaneous ICH includes blood pressure management, anti-epileptic drugs, and hemostasis. If the patient has coagulopathy or is undergoing anticoagulant therapy, correction with coagulopathy is necessary. Evaluation of surgical intervention is important, including in patients with a Glasgow Coma Scale (GCS) score ≤8, evidence of transtentorial herniation, or significant intraventricular hemorrhage or hydrocephalus. These factors should be considered in ICP monitoring and further surgical treatment. The removal of intracranial hematomas has many clinical benefits, such as the prevention of damage to the brain stem, cerebral herniation, controlled ICP management, and a decrease in excitotoxicity and neurotoxicity of blood products.^[3]^ Decompressive craniectomy with hematoma evacuation may play a role in comatose patients with significant midline shift and large hematomas on brain computerized tomography (CT) scans or patients with refractory increased ICP,^[4]^ which is defined as ICP > 20 mm Hg for >15 minutes in a 1-hour period refractory to first-tier therapies, surgical decompression is suggested. External ventricular drainage (EVD) is the procedure of choice for the treatment of acute hydrocephalus and increased ICP in patients with intracerebral hemorrhage and intraventricular hemorrhage.^[5]^ After initial operative management, the patient was admitted to an intensive care unit. There are many complications of spontaneous ICH, such as cerebral edema, rebleeding, seizure attacks, and hydrocephalus. Hydrocephalus is a common condition after spontaneous ICH, and post-hemorrhage is the second most common cause of non-obstructive hydrocephalus, especially in patients who undergo decompressive craniectomy (risk factor for hydrocephalus in patients with brain injury).^[6]^ The patients with intraventricular hemorrhage have a higher risk of developing permanent hydrocephalus and requiring shunting operations.^[7]^ However, there are no efficient risk factors for predicting the incidence of hydrocephalus following spontaneous ICH status after decompressive craniectomy.

Other risk factors for hydrocephalus in patients who have undergone decompressive craniectomy for spontaneous ICH have not been reported. Here, we retrospectively analyzed data from patients who underwent decompressive craniectomy for spontaneous ICH to identify the risk factors for postoperative hydrocephalus.

## 2. Materials and Methods

**Figure 1.**
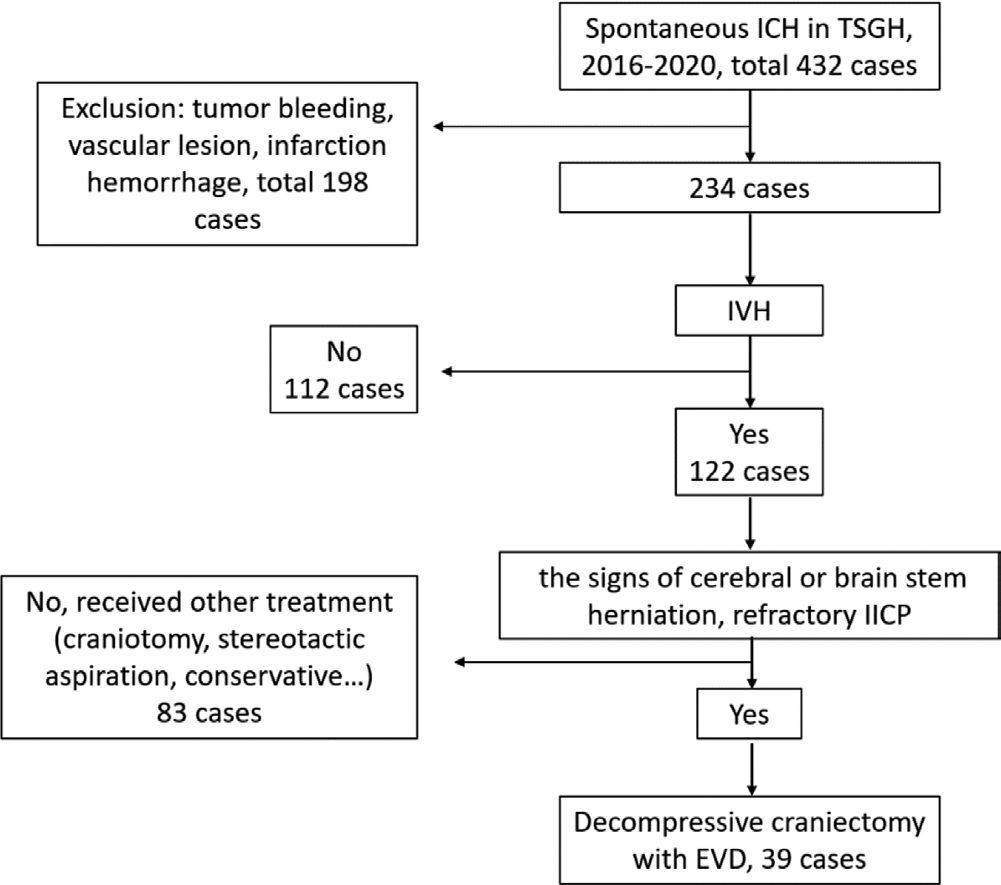
The flow chart of study designs.

**Figure 2.**
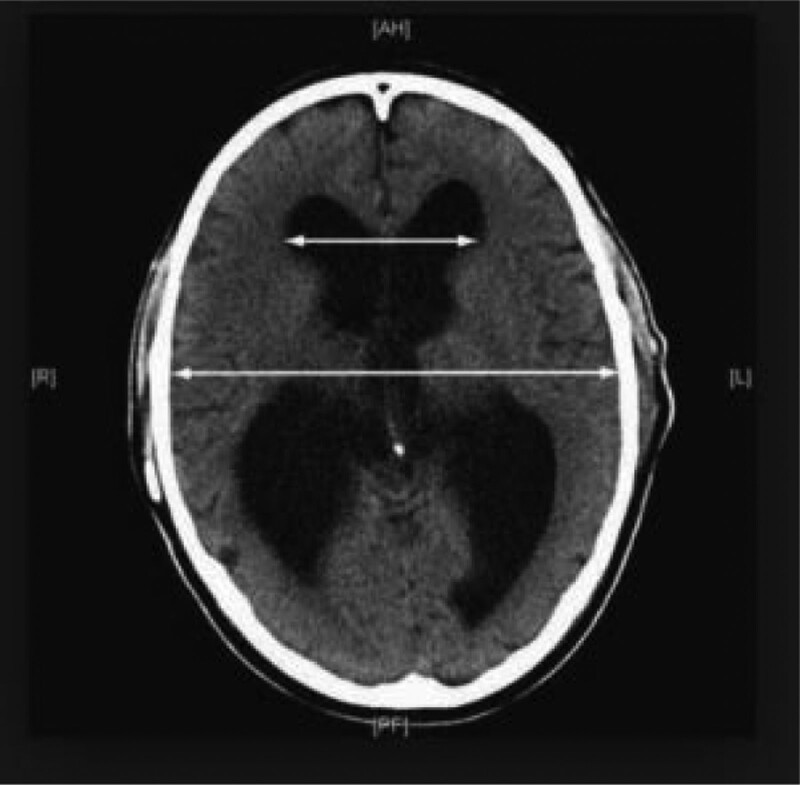
Evans ratios, the ratio of maximum width of the frontal horn to the maximum width of the inner table of the cranium recorded on the side contralateral to the decompressive craniectomy.

**Figure 3.**
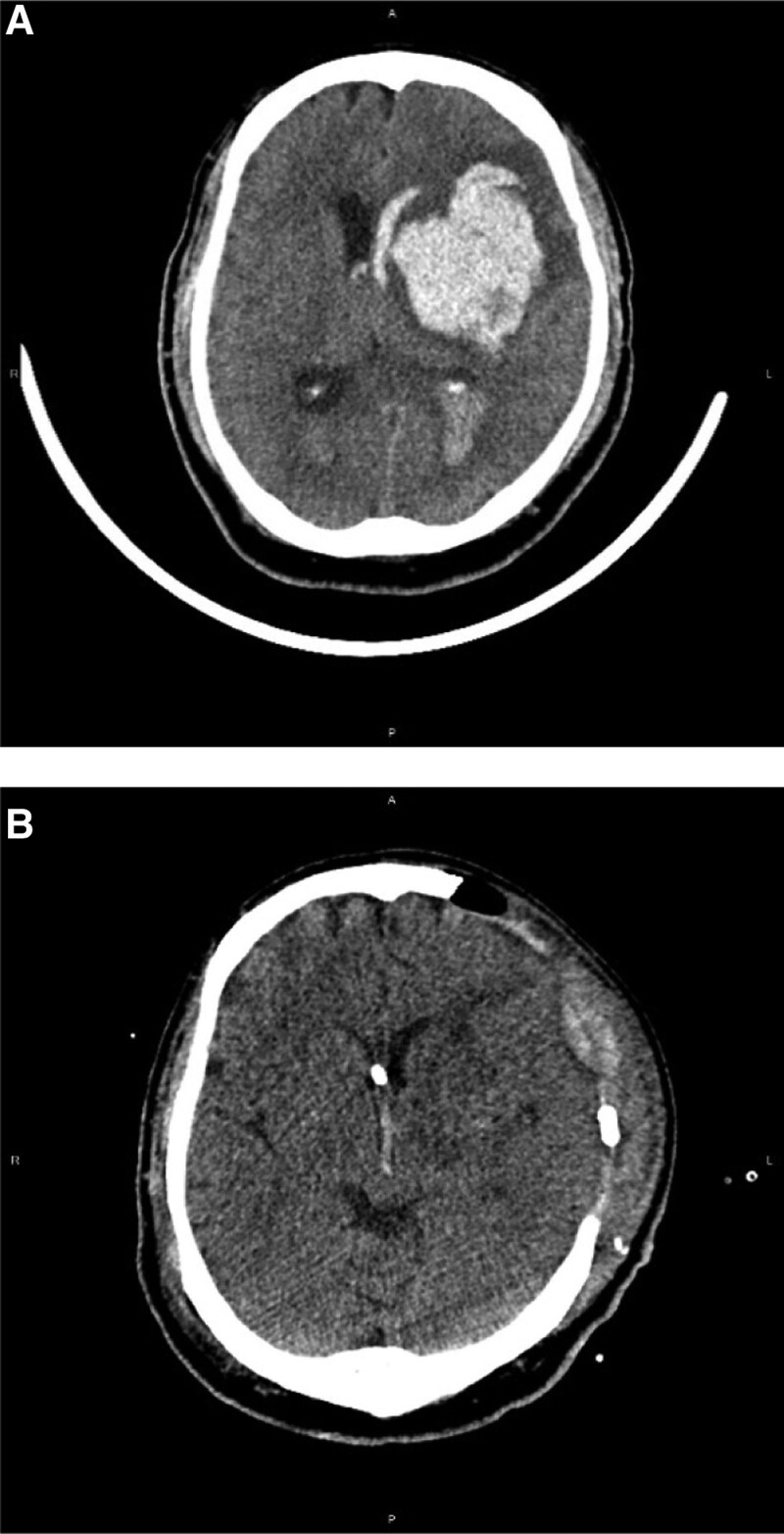
(A) The 20^th^ patient in our study, is a 66-year-old male, had a GCS of E2M4V2 when he was arrived to hospital. He suffered from spontaneous ICH with brain stem herniation over the left basal ganglion and intraventricular hemorrhage. (B) He received decompressive craniectomy and EVD after he was transferred to our hospital or about 445 min later. Brain CT scan on the 7^th^ day after operation showed nearly complete hematoma evacuation and no hydrocephalus after we clamped the EVD. We then removed the EVD on the 13^th^ day after operation. The GCS showed E3M6VT (T: tracheostomy) when he was transferred to the ordinary ward; the patient didn’t have symptoms of hydrocephalus on further follow up. CT = computerized tomography, EVD = external ventricular drainage, ICH = intracranial hemorrhage, GCS = Glasgow Coma Scale.

We studied patients with spontaneous intracranial hematoma with intraventricular hemorrhage and ventricular extension, suspicious acute hydrocephalus and mass effect who received decompressive craniectomy and EVD for medically refractory increased ICP at Tri-Service General hospital, Taipei, Taiwan from January 2016 to December 2020; 39 patients were included. Due to different pathophysiologies, pressure dynamics, and neurologic symptoms and signs of hydrocephalus in supra- and infra-tentorial hemorrhage, we only included patients with supratentorial hemorrhage. A flow chart of the study design is shown in Figure 1. Patient data were collected in accordance with the tenets of the Declaration of Helsinki. This retrospective study was approved by the institutional review committee of the Tri-Service General Hospital. Other patients were excluded for the following reasons: tumor bleeding, post-infarct hemorrhagic transformation, or death due to cardiopulmonary disorders. All patients were monitored in the intensive care unit and received medical treatment and management for ICP control (head elevation by 30°, anti-epileptic medication, sedation, etc). Decompressive craniectomy was performed when the patients had increased ICP and signs of brain stem herniation and intraventricular hemorrhage on brain CT. The intracerebral blood clot was almost completely removed during the operation. Ventriculoperitoneal (VP) shunting was performed after the patients showed signs of hydrocephalus during hospitalization (failed weaning of external ventricular draining, conscious disturbance after removal of EVD, etc). Evidence of hydrocephalus included failed weaning from EVD. During hospitalization, we tried to clamp the EVD and intensively monitored the patient’s clinical feature and conscious status.^[8]^ Under the critical care of post decompressive craniectomy, we set the EVD at level of 10 cm H2O over the foramen of Monroe; in order to force cerebrospinal fluid (CSF) flow through EVD in patients with ICP elevations, EVD could temporarily be lowered to the levels between 0 and 5 cm H2O. We will try to wean the EVD based on the relative vital signs, neurological function, and stable intracranial pressure after 1 week. The EVD systems were gradually raised in 5 cm steps every 24 hour up to a final level of 25 cm H2O, provided that it was clinically well tolerated. In case of successful weaning, EVD was subsequently closed for 48 hour. If the patient cannot tolerate the treatment, they may suffer from conscious disturbance, seizure, or other neurological deficits, and a VP shunt is suggested. The weaning period was approximately 2 weeks.^[9]^ Radiographic data on serial brain CT of ventricular dilation included the frontal horns, temporal horns, and third ventricle. A combination of unilateral ventricular dilation due to encephalomalacia and a normal-sized contralateral ventricle was defined as ventriculomegaly versus hydrocephalus. An Evans ratio of at least 0.3 may be consistent with a diagnosis of hydrocephalus (Fig. 2).^[10]^ The case of presentation of our clinical courses is in Figure 3. Clinical data and a series of brain CT scans for each patient were collected, as shown. The clinical data included patient age, sex, timing of operation, initial GCS, ICH score, alcohol consumption, cigarette smoking, hemodialysis, anticoagulant agent usage, history of cancer, previous stroke, heart valve diseases, type 2 diabetes mellitus, C-reactive protein (CRP), and albumin levels.

Univariate analysis was conducted to identify the association of each explanatory factor with the condition of hydrocephalus after intracranial hemorrhage (i.e., with or without hydrocephalus) using Student t test and the chi-square test with Fisher exact test for continuous and categorical data, respectively. Logistic regression was employed to determine predictors in the multivariate analysis, as described previously. A *P* value of .05 or less indicated a significant statistical difference.

## 3. Result

A total of 39 patients underwent decompressive craniectomy for spontaneous ICH with cerebral herniation. Indications for decompressive craniectomy in our study included emergency conditions with herniation or deteriorating symptoms. This study included 14 women and 25 men with spontaneous ICH. The baseline patient characteristics are presented in Table 1. The patients’ ages ranged from 35 to 79 years (mean: 58.56 years). The mean spontaneous ICH volume was 66 mL. The delay from the emergency department to craniectomy varied: the hydrocephalus group was 112 to 552 minutes (mean: 232.12 minute), and the non-hydrocephalus group was 107 to 969 minutes (mean: 371.5 minute). Postoperative hydrocephalus developed on serial brain CT scans in 17 patients after decompressive craniectomy. The duration from craniectomy to ventriculoperitoneal shunting (VP shunting) ranged from 6 to 30 days (mean: 14.5 days). The demographic data of the 39 patients who underwent decompressive craniectomy due to spontaneous ICH are summarized in Table 2. The average follow-up period of our patients was approximately 12 weeks, and we plan to arrange cranioplasty after adequate clinical conditions. No patient developed hydrocephalus at a later time point.

**Table 1:** The baseline characteristics of the patients.

Variables	Mean	Minimal	Maximal
Age (yrs)	57.78	24	89
hematoma volume (mL)	66.0	38.8	84.0
Time to operating room (min)	310.74	107	969
Time to VP shunting (days)	14.5	6	30
Albumin	3.41	2.2	4.3
CRP	3.48	0.12	9.44

CRP = C-reactive protein, VP shunting = ventriculoperitoneal shunting.

**Table 2:** Demographic data of 39 patients with decompressive craniectomy due to spontaneous ICH.

No.	Age	Gender	Time to OP (min)	Blood volume (mL)	MLS (mm)	Pre-DC GCS	Drink	Smoke	Comor-bidity (total)	Hydro-cephalus
1	54	Male	225	77.2	6.2	E1M2V1	Y	Y	6	N
2	64	Female	228	72.8	5.5	E3M5V1	N	N	2	N
3	68	Female	410	61.0	4.0	E1M3V1	N	N	4	N
4	68	Male	163	72.0	3.8	E3M6V3	N	Y	1	N
5	47	Male	552	60.3	5.2	E4M5V3	Y	Y	3	Y
6	74	Male	130	58.5	4.2	E3M5V2	N	N	2	N
7	51	Female	347	84.0	7.1	E1M1V1	N	N	2	N
8	48	Male	453	72.6	6.0	E3M5V2	Y	Y	2	Y
9	53	Male	236	49.7	3.6	E4M5V2	N	N	1	N
10	75	Male	172	38.8	4.7	E1M4V1	N	Y	2	Y
11	59	Male	197	53.3	4.2	E3M5V1	N	N	3	N
12	50	Male	519	56.2	4.4	E3M5V3	N	N	1	N
13	79	Female	336	74.6	6.3	E1M2V1	Y	Y	2	N
14	71	Male	329	61.0	4.8	E1M4V1	N	Y	2	N
15	62	Female	148	52.0	4.5	E3M6V3	N	N	5	Y
16	35	Male	192	82.2	7.0	E2M4V1	Y	Y	1	Y
17	63	Male	189	60.8	4.2	E1M2V1	N	Y	3	Y
18	48	Male	229	52.4	4.4	E3M5V4	N	Y	2	Y
19	51	Male	307	83.8	7.4	E1M1V1	Y	Y	1	Y
20	66	Male	445	68.6	6.0	E2M4V2	N	Y	1	N
21	35	Female	158	53.5	4.0	E2M4V1	N	N	0	Y
22	73	Male	122	82.2	6.8	E2M4V1	Y	Y	1	Y
23	43	Male	309	75.4	4.6	E2M5V2	N	N	1	N
24	61	Female	265	48.8	4.8	E2M4V1	N	N	2	Y
25	67	Female	134	48.1	4.3	E1M1V1	N	Y	1	Y
26	57	Male	250	65.3	5.3	E1M2V1	N	N	2	N
27	56	Male	112	78.8	5.6	E4M5V3	Y	Y	1	Y
28	36	Male	426	68.7	4.3	E2M4V1	N	N	2	N
29	70	Female	227	80.5	5.5	E2M4V2	N	N	1	Y
30	57	Female	107	76.6	6.2	E2M5V2	N	N	2	Y
31	53	Male	969	80.2	6.8	E1M2V1	N	N	1	N
32	52	Male	457	79.6	6.0	E4M5V3	N	N	2	N
33	55	Female	139	77.0	5.8	E2M5V1	N	N	4	Y
34	73	Female	443	75.2	5.2	E4M5V3	N	N	3	N
35	78	Male	701	82.8	6.5	E1M2V1	Y	Y	5	N
36	50	Female	492	82.9	6.2	E4M5V3	N	N	1	N
37	67	Male	344	79.4	6.6	E1M1V1	Y	Y	2	N
38	63	Female	255	64.5	4.0	E1M2V1	N	N	2	N
39	52	Male	177	62.7	4.2	E1M1V1	N	Y	2	Y

Drink = alcohol drinking, GCS = Glasgow Coma Scale, ICH = intracranial hemorrhage, MLS = midline shift, OP = operation, Pre-DC = pre-decompressive craniectomy, smoke = cigarette smoking. Comorbidity, including hemodialysis, hypertension, Anticoagulant usage, heart valve diseases, previous stroke, type 2 diabetes mellitus, hyperlipidemia. Y: yes, N: no.

The development of postoperative hydrocephalus was not significantly associated with patient age, sex, GCS score on admission, ICH score, hemodialysis, anticoagulant agent usage, history of cancer, previous stroke, heart valve diseases, type 2 diabetes mellitus, CRP, and albumin (Table 3). However, the timing of surgery, cigarette smoking, and alcohol consumption were significantly associated with postoperative hydrocephalus (Table 2). Univariate and multivariate logistic regression analyses were used to evaluate the data presented in Table 4. Patients who underwent ultra-early operation (less than 4 hours) were likely to have a higher incidence of hydrocephalus than those who underwent an operation more than 4 hours ago (*P* valve: .038; odds ratio (OR) 6.79, 95% confidence ratio (CI) 1.19–38.57). Patients who smoked cigarettes were associated with postoperative hydrocephalus (*P* valve: .021; OR, 14.27; 95% CI:1.12–181.79).

**Table 3:** The distribution of demography and clinical characteristic by treatment.

	Without hydrocephalus (n = 22)	With hydrocephalus (n = 17)	Without hydrocephalus vs with hydrocephalus *P value
Age, n (%)			.606†
>=65	7(53.80)	6(46.20)	
35-64	15(60.0)	10(40.0)	
<35	0(0)	1(100.0)	
Gender, n (%)			.789
Female	7(50.0)	7(50.0)	
Male	15(60.0)	10(40.0)	
GCS on admission, n (%)			.508†
>=12	3(42.9)	4(57.1)	
5–11	12(54.5)	10(45.5)	
< 5	7(70.0)	3(30.0)	
ICH score, M ± SD	2.91 ± 0.75	2.82 ± 0.81	.260
Time to operation n (%)			.038
>4 h	15(75.0)	5(25.0)	
<4 h	7(36.8)	12(63.2)	
Alcohol drinking, n (%)			.033†
No	19(67.9)	9(32.1)	
Yes	3(27.3)	8(72.7)	
Cigarette smoking, n (%)			.021
No	17(73.9)	6(26.1)	
Yes	5(31.3)	11(68.8)	
Hemodialysis, n (%)			.618†
No	19(54.3)	16(45.7)	
Yes	3(75.0)	1(25.0)	
Hypertension, n (%)			.147†
No	1(20.0)	4(80.0)	
Yes	21(61.8)	13(38.2)	
Anticoagulant, n (%)			.464†
No	15(51.7)	14(48.3)	
Yes	7 (70.0)	3(30.0)	
Heart valve diseases, n (%)			1.000†
No	20(55.6)	16(44.4)	
Yes	2(66.7)	1(33.3)	
Previous stroke, n (%)			.438†
No	17(53.1)	15(46.9)	
Yes	5(71.4)	2(28.6)	
Type 2 diabetes mellitus, n (%)			.465†
No	18(60.0)	12(40.0)	
Yes	4(44.4)	5(55.6)	
Hyperlipidemia, n (%)			.568
No	16(61.5)	10(38.5)	
Yes	6(46.2)	7(53.8)	
Midline shift on brain CT (>0.5 cm), n (%)			.823
No	11(61.1)	7(38.9)	
Yes	11(52.4)	10(47.6)	
Albumin, n (%)			.129
<3.5	9(42.9)	12(57.1)	
>=3.5	13(72.2)	5(27.8)	
CRP, n (%)			1.000
<1.0	7(53.8)	6(46.2)	
>1.0	15(57.7)	11(42.3)	

CT = computerized tomography, CRP = C-reactive protein, GCS = Glasgow Coma Scale, M ± SD = mean ± deviation, spontaneous ICH = spontaneous intracranial hemorrhage.

* Mann–Whitney U test or chi-square test.

† Fisher exact test.

**Table 4:** Univariate and multivariate of logistic regression analysis.

Multivariate			
Variables	OR	(95% CI)	*P* value
Age (>=65 yrs vs <65 yrs)	0.60	(0.11–3.42)	.566
Sex (male vs female)	0.08	(0.01–0.99)	.049
Timing of operation (<4 h vs ≥4 h)	6.79	(1.19–38.57)	.031
Alcohol drinking	4.14	(0.43–39.42)	.217
Cigarette smoking	14.27	(1.12–181.79)	.041

CI = confidence interval, OR = odds ratio, ref = reference group.

## 4. Discussion

Our study aimed to determine the predictors of hydrocephalus in patients with spontaneous ICH. We found at least 4 predictors for a high risk of hydrocephalus: timing of operation, cigarette smoking, and alcohol drinking. These factors were statistically significant in predicting the incidence of hydrocephalus.

We reviewed the literature on hydrocephalus and decompressive craniectomy. Some studies hypothesized that the abnormal collections of hygromas, hydrocephalus, and subgaleal hygromas after decompressive craniectomy are caused by altered brain pulsatility, CSF hydrodynamics, decreased cerebral blood flow, and impaired brain glymphatic clearance in a vulnerable subset of patients..^[11]^ Extension to the ventricles was the only independent risk factor for hydrocephalus (4–13 days), while extension to ventricles, decompressive craniotomy, and intracranial infection were independent predictors of hydrocephalus (≥14 days).^[12]^ However, we found that some patients who underwent decompressive craniectomy due to spontaneous ICH had a higher risk of hydrocephalus. Another study suggested a link between decompressive craniectomy and hydrocephalus in the setting of traumatic brain centers on the intracranial dura-arachnoid interface, where shearing forces from the primary injury may critically interrupt CSF resorption systems.^[13]^ When such a disruption is followed by the characteristically large craniectomy required for trauma management, the abnormal resulting transcerebral and intracranial pressure gradients allow for a marked expansion of all the subdural spaces given the pressure-dependent nature of the proposed mechanism.^[13]^

We attempted to explain the association between cigarette smoking or alcohol consumption and the higher risk of postoperative hydrocephalus. There is no strong evidence that smoking history or alcohol consumption is associated with a higher risk of hydrocephalus following decompressive craniectomy in spontaneous ICH. This may be associated with the side effects of ethanol, nicotine, or tar oil. However, further studies are required for further evaluation. According to the literature, smoking is associated with cerebrospinal fluid shunt in patients with idiopathic intracranial hypertension (Investigative Ophthalmology & Visual Science, 2013), It was also found that smokers had a greater odds of undergoing cerebrospinal fluid shunt compared to nonsmokers. The reason for this remains unclear. Although the mechanism of smoking and hydrocephalus is still unclear, we can also inform patients that smoking may be a risk factor for hydrocephalus after intracranial hemorrhage and encourage them to quit smoking.

**Figure 4.**
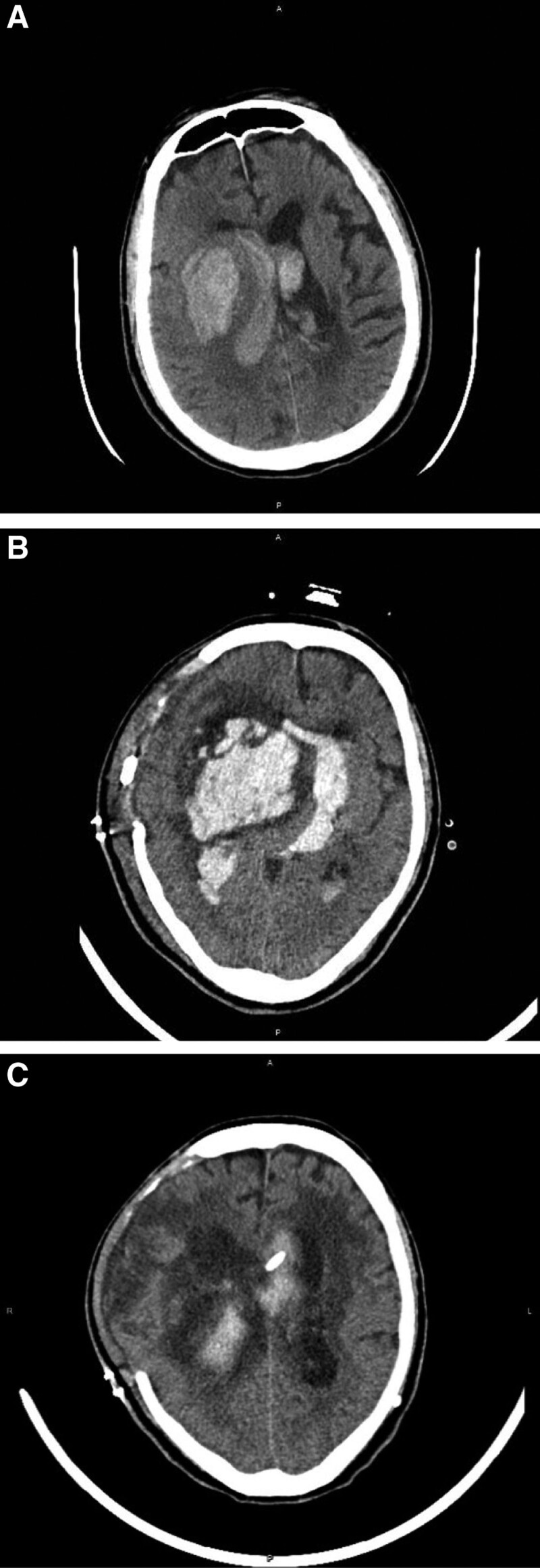
(A) The 22nd patient in our study, is a 73-year-old male with GCS of E2M4V1 when he was arrived to hospital, suffered from spontaneous right putamen ICH. (B) He received decompressive craniectomy and EVD, and the timing of the operation was about 122 min. However, he was still in a coma, and repeated brain CT data showed a rebleeding hematoma on day 3. (C) The patient was received reoperation of removal of hematoma and check bleeding. After the operation, his GCS showed improved (E1M2VT to E2M4VT). The patient suffered from worsening neurological symptoms after we clamp the EVD tube. The following brain CT revealed persistent hydrocephalus with dilated lateral ventricle; he then received the VP shunting operation. CT = computerized tomography, EVD = external ventricular drainage, ICH = intracranial hemorrhage, GCS = Glasgow Coma Scale, VP shunting = ventriculoperitoneal shunting.

The group that required more than four hours after spontaneous ICH symptoms to reach the operating room had a lower incidence of hydrocephalus. We sometimes need to arrange the operation as soon as possible because of significant brain stem herniation or refractory increased ICP, but there is higher evidence of hydrocephalus in this group. According to the journal, rebleeding leads to worse outcomes in ultra-early craniotomy for spontaneous ICH.^[14]^ Rebleeding was more common in patients who underwent surgery within four hours compared with 12 hours.^[14]^ The 22th patient is a case who experienced rebleeding and underwent reoperation for hematoma removal due to significant mass effect and neurological deficit (Fig. 4). There was a relationship between rebleeding and mortality in the 4-hour surgery group. More rebleeding might influence hydrocephalus in follow-up studies; we surveyed the residual intracranial blood volume postoperatively following brain CT. More residual hematoma volumes after surgery have a significant association with hydrocephalus following decompressive craniectomy, which may be linked to a higher rebleeding rate after surgery. However, further evidence is required for further evaluation.

Our study has some limitations. A major limitation of our study is that it was retrospective non-randomized and single. These factors can lead to information bias owing to unclear data collection. Second, we did not explain all of the results; they need to be evaluated further to understand mechanisms such as rebleeding with hydrocephalus and the influence of ethanol, nicotine, and tar oil. Therefore, more clinical studies are needed to explore the effectiveness of this treatment in patients who undergo decompressive craniectomy. Another limitation is that we excluded patients who died after decompressive craniectomy. The patient who expired during the postoperative care may have multiple possible factors, such as infections, infarction, cerebrovascular failure, or the effect of hydrocephalus. We cannot easily distinguish these complex possibilities, and we need to research more evidence and modify our study to improve it. However, we did not discuss the relationship between hydrocephalus and postoperative complications of decompressive craniectomy, such as infection, pneumocephalus, or sinking flap syndrome. Rebleeding was the only post-operative complication that we studied. This is our limitation and we will evaluate this issue in future studies.

## 5. Conclusion

Hydrocephalus is a complication in patients who are suffered from spontaneous ICH with brain stem herniation. It most commonly occurs at the onset of spontaneous ICH. Ultra-delayed operation (more than 4 hours) and non-cigarette smoking showed a relatively lower risk of postoperative hydrocephalus. Physicians should remember the higher incidence of postoperative hydrocephalus before ultra-early operation for spontaneous ICH. Patients suffering from spontaneous ICH with brain stem herniation need to go to the operating room as soon as possible for decompressive craniectomy, but physicians should remind patients and their families with a higher incidence of postoperative hydrocephalus. Therefore, VP shunting may be necessary in the future.

## Author contributions

Yi-Chieh Wu, Jang-Chun Lin, Yu-Ching Chou and Wei-Hsiu Liu was responsible for designing this research. Hsiang-Chih Liao, Da-Tong Ju, Dueng-Yuan Hueng, Chi-Tun Tang, Kuan-Yin Tseng, Kuan-Nien Chou, Bon-Jour Lin and Shao-Wei Feng extracted the data and conducted the statistical analysis. Yi- An Chen, Ming-Hsuan Chung and Peng- Wei Wang drafted the manuscript.
